# Retrospective Evaluation of Prognostic Variables and Overall Survival Associated With Nonketotic Hyperosmolar Hyperglycemia in Diabetic Cats: 29 Cases (2000–2020)

**DOI:** 10.1111/vec.70025

**Published:** 2025-08-29

**Authors:** Annalisa Judy, Aaron Rendahl, Kelly Tart

**Affiliations:** ^1^ University of Minnesota College of Veterinary Medicine St. Paul Minnesota USA

**Keywords:** diabetes mellitus, DKA, endocrine emergency, feline, HHS, hyperosmolality

## Abstract

**Objective:**

To compare survival between cats diagnosed with a nonketotic hyperosmolar hyperglycemic state (HHS) and cats diagnosed with diabetic ketoacidosis (DKA), and to determine whether clinical parameters, clinicopathologic data, and insulin type are associated with survival. Secondary objectives were to evaluate whether these parameters were associated with survival in cats undergoing a generalized hyperglycemic diabetic crisis.

**Design:**

Retrospective evaluation of medical records of cats diagnosed with DKA and HHS between 2000 and 2020.

**Setting:**

University teaching hospital.

**Animals:**

The HHS group consisted of 29 cats (blood glucose concentration [BG] ≥31.08 mmol/L [≥560 mg/dL]; calculated serum osmolality ≥350 mOsm/kg; ketone negative). The DKA group consisted of 71 cats (BG ≥7.77 mmol/L [≥140 mg/dL]; pH <7.35; ketone positive).

**Interventions:**

None.

**Measurements and Main Results:**

No clinicopathologic parameters or previous use of glargine U‐100 were significantly associated with survival in the HHS group. There was no difference in survival of the HHS group versus the DKA group (65.5% vs. 68.6%; *p* = 0.85). In all cats, higher serum BUN (*p* = 0.014), creatinine (*p* = 0.0098), or BG (*p* = 0.015) and lower serum sodium concentration (*p* = 0.03) or body temperature (*p* = 0.0098) were associated with decreased odds of survival. Calculated total and effective osmolalities were not associated with survival in either group.

**Conclusions:**

Cats with HHS had a survival of 65.5% in this study. No serum biochemical parameters predicted prognosis for the HHS group, and insulin type was not found to be associated with survival. Azotemia, hyponatremia, hyperglycemia, and hypothermia were associated with decreased survival when combining all cats. Hyperosmolality was not associated with survival; therefore, distinguishing HHS and DKA into separate syndromes may be arbitrary.

**Abbreviations**
AKI: acute kidney injury
BCS: body condition score
BG: blood glucose concentration
CI: confidence interval
DKA: diabetic ketoacidosis
DM: diabetes mellitus
GFR: glomerular filtration rate
HHS: hyperosmolar hyperglycemic state
IQR: interquartile range
OR: odds ratio

## 1 Introduction

Two recognized hyperglycemic diabetic crises requiring emergency care in cats include diabetic ketoacidosis (DKA) and hyperosmolar hyperglycemic state (HHS). Historically, HHS has been called hyperosmolar hyperglycemic syndrome or hyperosmolar hyperglycemic nonketotic syndrome, and has been traditionally defined as a blood glucose concentration (BG) >30–33.3 mmol/L (>540–600 mg/dL) and serum osmolality >350 mOsm/kg [1, 2]. HHS has traditionally been considered a separate and more life‐threatening syndrome in cats and is often coupled with severe dehydration and total body potassium depletion, and the most severely affected patients can present in a comatose state [1, 2]. In dogs, HHS has been associated with 62% survival, while in cats, HHS has been associated with very high mortality [3]. A retrospective study evaluating 17 cats diagnosed with HHS reported a 35.3% survival to discharge and long‐term survival of 12% [1]. DKA is more common than HHS and is associated with metabolic alterations such as metabolic acidosis and ketonemia [2].

The pathophysiology of HHS is similar to that of DKA in that there is a partial or relative insulin deficiency that reduces cellular glucose uptake. There may be a component of insulin resistance that is promoted by concurrent infection, inflammation, or organ failure, causing an increased secretion of counterregulatory hormones [4, 5, 6, 7, 8]. Patients with HHS produce fewer ketone bodies than those with DKA. This is believed secondary to a small remainder of functional pancreatic beta cells secreting trace insulin: enough to prevent lipolysis and ketogenesis, but not enough to utilize glucose and prevent hyperglycemia [4, 9, 10]. The glomerular filtration rate (GFR) is notably decreased in HHS due to marked dehydration and hypovolemia, which reduces the kidneys’ ability to excrete excess glucose and subsequently causes marked hyperglycemia and dehydration [11, 12]. Cerebral dehydration secondary to severe hyperglycemia can cause profound neurologic abnormalities such as decreased mentation, severe weakness, miosis, abnormal cranial nerve reflexes, seizures, or coma, which distinguishes these cases from those with DKA or stable diabetes mellitus (DM) [1]. Approximately 30% of people presenting in a diabetic crisis display clinical features of both DKA and HHS, indicating these syndromes may be interrelated rather than separate conditions [7].

While HHS has been described in veterinary medicine, there are limited studies characterizing this condition in cats. A 2004 study by Koenig et al. [1] that evaluated HHS in a population of 17 cats is the most comprehensive study to date. This study found that cats with HHS were more likely to be hypernatremic, normokalemic, hyperglycemic, and hyperosmolar in comparison to cats with DKA or DM. Cats with HHS were found to have serious comorbidities, including chronic kidney disease, infectious disease, and congestive heart failure. This study was unable to identify specific prognostic factors for HHS; however, a low survival of 35.3% was noted. To the authors’ knowledge, no further comprehensive studies have been performed regarding this condition in cats.

The primary objectives of this study were to compare signalment, presenting clinical parameters, medical history, and clinicopathologic data of cats with HHS to cats with DKA; document the overall survival of cats diagnosed with HHS; and analyze variables that may be associated with survival in cats diagnosed with HHS. Secondary objectives were to investigate whether the use of glargine U‐100^a^ insulin was associated with survival and to evaluate for parameters associated with survival for all diabetic cats experiencing a hyperglycemic diabetic crisis. The authors hypothesized that cats diagnosed with HHS would have poorer survival than cats with DKA and that cats with HHS presenting with hypothermia, azotemia, and normokalemia would have poorer survival than cats without those abnormalities.

## 2 Methods

### 2.1 Data Collection

The records of all cats diagnosed with DM and DKA that presented to University of Minnesota College of Veterinary Medicine's veterinary teaching hospital between 2000 and 2020 were accessed and reviewed to distinguish cases diagnosed with HHS, with DKA, or with neither. A total of 810 records of diabetic cats admitted to the hospital were reviewed and categorized. Cats were tentatively included in the HHS group if they had calculated serum osmolality concentration ≥350 mOsm/kg, BG ≥31.08 mmol/L (≥560 mg/dL), and no ketones present in any serum or urine tested. Ketone bodies were either measured as beta‐hydroxybutyrate concentration in serum or acetoacetic acid detected via urine dipstick. Cats were tentatively included in the DKA group if their BG was ≥7.77 mmol/L (≥140 mg/dL) or there was a history of previously diagnosed DM; blood pH was ≤7.35; and ketones were present via beta‐hydroxybutyrate concentration in serum or acetoacetic acid detected via urine dipstick [13].

Thirty‐five cases met the tentative inclusion criteria for the HHS group and 182 fit the tentative inclusion criteria for the DKA group. Six cats were then eliminated from the HHS group due to incomplete medical records; thus, 29 cases were ultimately included in the HHS group. Of 182 cases fitting the tentative inclusion criteria for DKA, 71 cases were randomly selected and ultimately included to form the DKA group; this case number was chosen as twice the number of cases tentatively included in the HHS group. The DKA group served as the control group for this study.

Medical records were reviewed for signalment, presenting mentation, body weight, body condition score (BCS; 1–9), temperature, heart rate, and respiratory rate [14]. The definitions associated with mentation included bright and alert, quiet and alert, dull, obtunded, stuporous, and comatose. Additional information collected from the medical records included concurrent and historic comorbidities, previously diagnosed DM versus first presentation, administered insulin type for established diabetics, initial blood work (CBC^b^, serum biochemistry panel^c^, and venous blood gas^d^), urinalysis findings, and whether the patient survived to discharge. Clinicopathologic data recorded included HCT and WBC count; serum BUN, creatinine, sodium, potassium, chloride, phosphorus, total calcium, magnesium, BG, total bilirubin, albumin, and total serum concentrations; serum alkaline phosphatase, gamma‐glutamyl transferase, alanine transaminase, and aspartate aminotransferase activities; total and effective serum osmolalities; and urine specific gravity. Serum sodium concentration recorded was not corrected for the degree of hyperglycemia. Total serum osmolality was calculated based on values found on the serum biochemistry panel^c^. Total serum osmolality was calculated as follows: Osmolality_T_ = 2(Na + K) + BUN (mg/dL)/2.8 + BG (mg/dL)/18 [15]. Effective osmolality was calculated as follows: Osmolality_E_ = 2(Na) + BG (mg/dL)/18 [1]. The upper limits of BUN and BG on venous blood gas panels^d^ are 49.98 mmol/L (140 mg/dL) and 38.85 mmol/L (700 mg/dL), respectively. Therefore, if BUN or BG values exceeded these parameters on venous blood gas panels, they were assigned as BUN = 49.98 mmol/L (140 mg/dL) or BG = 38.85 mmol/L (700 mg/dL), respectively. In addition to comparing the collected data between the HHS and DKA groups, the following variables were compared between survivors and nonsurvivors of the entire cohort: rectal temperature, BCS, previous use of glargine, concurrent disease, total serum osmolality, effective serum osmolality, and serum concentrations of BUN, creatinine, sodium, potassium, phosphorus, and BG. The pooled group of cats experiencing a hyperglycemic diabetic crisis is referred to as the “combined group” throughout the paper. Mortality was defined as either survival or nonsurvival to hospital discharge. Nonsurvivors included patients that experienced natural death or euthanasia. The reason for euthanasia was noted to be from progression of condition despite treatment, poor prognosis, or unknown.

### 2.2 Statistical Methods

To describe the DKA and HHS groups, counts and percentages were reported for categorical variables of interest, and medians and interquartile ranges (IQRs) were reported for numerical variables of interest. Differences were tested using Fisher's test for categorical variables and Wilcoxon's test for numerical variables, and *p*‐values are reported. All *p*‐values reported were adjusted for multiple comparisons using the Benjamini–Hochberg adjustment for false discovery rate; this was performed for all DKA versus HHS comparisons together, and for survival risk factors for the DKA, HHS, and combined groups separately. A *p*‐value of <0.05 was considered significant.

To explore the selected risk factors of interest, individual logistic regression models were fit with survival as the response variable and each risk factor in turn as the predictor; these were fit for the DKA, HHS, and combined groups separately. The standardized odds ratio (OR) for each risk factor, along with 95% confidence intervals (CIs) and *p*‐values, were reported, along with counts and percentages for categorical variables and medians and IQRs for numerical variables. To standardize the OR, the standard deviation of all individuals was used for models for all three groups so that the size of the effects was comparable among the groups. All variables were also checked for normality using boxplots; creatinine was seen to be right‐skewed and so was log‐transformed before modeling.

## 3 Results

### 3.1 Signalment

In the 71 cats that comprised the DKA group, there were 50 domestic shorthair, nine domestic longhair, five Siamese, three domestic medium hair, and one each of Birman, Maine Coon, American Bobcat, and Russian Blue. Median age at admission for cats with DKA was 11 years (IQR: 8–14), with a range of 6 months to 19 years. There were 25 spayed females and 46 neutered males. In the 29 cats comprising the HHS group, there were 23 domestic shorthair, two domestic longhair, two Manx, and one each of domestic medium hair and Siamese. The median age of cats with HHS at presentation was 14 years (IQR: 12.8–15), with a range of 6–20 years. There were 11 spayed females, one intact female, and 17 neutered males. The median age of cats in the HHS group was older than cats in the DKA group (*p* = 0.0038).

### 3.2 Presenting Clinical Parameters

In the DKA group, 14% (10/71) presented bright and alert, 29.6% (21/71) presented quiet and alert, 29.6% (21/71) presented with a dull mentation, and 22.5% (16/71) presented obtunded. There were 4.2% (3/71) that had no mentation noted in their medical record. The median body weight of cats with DKA was 4.72 kg (IQR: 3.88–5.69 kg) with a median BCS of 5/9 (IQR: 4–7). Median temperature at presentation was 37.7°C (IQR: 36.8–38.6; 99.8°F [IQR: 98.3–101.4]), median heart rate was 170 bpm (IQR: 150–200), and median respiratory rate was 32 per minute (IQR: 30–44).

Of the cats with HHS, 3.4% (1/29) presented bright and alert, 27.6% (8/29) presented quiet and alert, 31.0% (9/29) presented with a dull mentation, and 13.8% (4/29) presented obtunded. No mentation was noted in the medical record of 24.1% (7/29) patients. The median body weight of the HHS cats was 4.3 kg (IQR: 3.17–5.31) with a median BCS of 4/9 (IQR: 2–7). Median body temperature was 37.6°C (IQR: 37.6–38.4; 99.6°F [IQR: 98.5–101.2]), median heart rate was 182 bpm (IQR: 178–200), and median respiratory rate was 30 per minute (IQR: 21–36). There were no significant differences in weight, temperature, heart rate, or respiratory rate between the DKA and HHS groups (Table 1).

**TABLE 1:** Summary of the median (IQR) for age, weight, BCS, and vital parameters for 71 cats with DKA and 29 cats with HHS.

	DKA	HHS	*p*‐value
Age (years)	**11 (8–14)**	**14 (12.8–15)**	**0.0038**
Weight (kg)	4.72 kg (3.88–5.69)	4.3 kg (3.17–5.31)	0.18
BCS	5 (4–7)	4 (2–6)	0.13
Temperature (°C, °F)	37.7°C (36.8–38.6) 99.8°F (98.3–101.4)	37.6°C (36.9–38.4) 99.6°F (98.5–101.2)	1.0
Heart rate (beats/minute)	170 (150–200)	182 (178–200)	0.21
Respiratory rate (breaths/minute)	32 (30–44)	30 (21–36)	0.074

Abbreviations: BCS, body condition score; DKA, diabetic ketoacidosis; HHS, hyperglycemic hyperosmolar state; IQR, interquartile range.

Significantly different values between the DKA and HHS group are **bolded**.

*p*‐value represents a *p*‐value with Benjamini–Hochberg correction.

### 3.3 Previous Medical History

In the DKA group, 57.7% (41/71) were established diabetics and the most common type of insulin used was glargine U‐100^a^ (53.7%, 22/41), followed by human recombinant protamine zinc insulin^e^ (17.1%, 7/41). Less common insulin types included lente^f^^,^ g (14.6%, 6/41), neutral protamine Hagedorn insulin^h^ (4.9%, 2/41), and unknown (9.8%, 4/41). The majority of cats in the DKA group (66.2%, 47/71) had no concurrent diseases noted at presentation. Of the 24 cats with DKA that had concurrent disease, the most common was gastrointestinal disease (12.7%, 9/71). The other concurrent diseases within the DKA group included kidney disease (11.3%, 8/71), cardiac disease (5.6%, 4/71), lower urinary tract disease (5.6%, 4/71), hyperthyroidism (3/71, 4.2%), hepatic disease (2.8%, 2/71), and seizures (1.4%, 1/71).

In the HHS group, 72.4% (21/29) had a previous diagnosis of DM and the most common type of insulin used was glargine U‐100^a^ (38.1%, 8/21), followed by human recombinant protamine zinc insulin^e^ (14.3%, 3/21), lente^f^^,^ g (14.3%, 3/21), and neutral protamine Hagedorn^h^ (9.5%, 2/21). The insulin type was unknown in 23.8% of cases (5/21). There were no concurrent diseases diagnosed in 31% (9/29) of the HHS group. Fifteen cats with HHS had concurrent disease, and the most common was kidney disease (34.4%, 10/29), followed by cardiac disease (6.9%, 2/29), neoplasia (6.9%, 2/29), and lower urinary disease (3.4% 1/29). Concurrent disease status was unknown in 17% (5/29) of the HHS group.

A higher percentage of cats in the HHS group had concurrent diseases than in the DKA group (HHS: 51.7%, DKA: 35.2%; *p* = 0.043). There was no difference in the percentage of cases that had previously diagnosed DM (HHS: 72.4%; DKA: 57.7%; *p* = 0.25) or cases maintained on glargine U‐100 at time of presentation (HHS: 27.6%; DKA: 31%; *p* = 0.85).

### 3.4 Clinicopathologic Data

**FIGURE 1:**
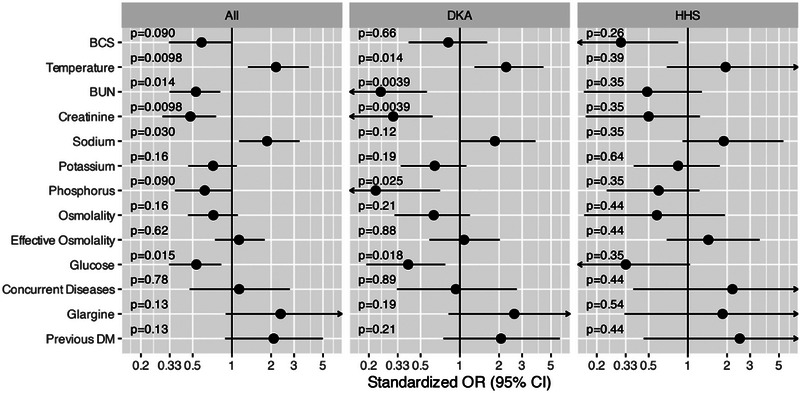
Standardized ORs and 95% CIs for survival of various clinical signs, presenting physical examination findings, and clinicopathologic parameters from 100 diabetic cats (“All”) experiencing a hyperglycemic diabetic crisis: 29 cats diagnosed with HHS and 71 diagnosed with DKA. Parameters with an OR and 95% CI to the left of the *y*‐axis had a negative association with survival, while parameters with OR and 95% CI to the right of the *y*‐axis had a positive association with survival. Parameters that cross the *y*‐axis did not have a significant association with survival (*p* > 0.05). For the combination group (“All”), low temperature, low sodium, increased BUN, increased creatinine, and increased glucose were associated with decreased survival. For the DKA group, low temperature, increased BUN, increased creatinine, increased glucose, and increased phosphorus were associated with decreased survival. No significant associations between parameters and survival were found with the HHS group. BCS, body condition score; CI, confidence interval; DKA, diabetic ketoacidosis; DM, diabetes mellitus; HHS, hyperosmolar hyperglycemic state; OR, odds ratio.

**FIGURE 2:**
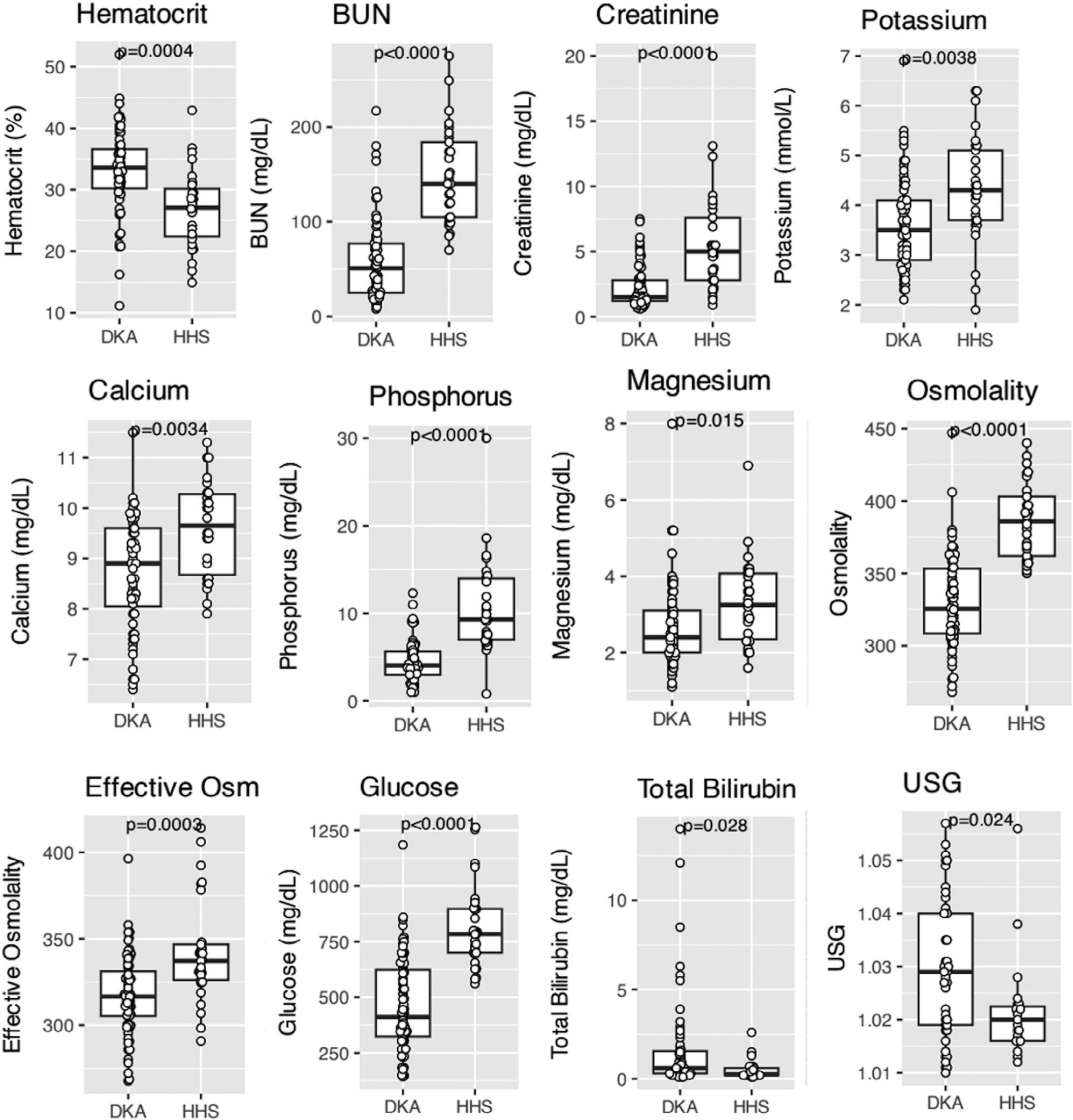
Dot plots representing the median (thick horizontal line), interquartile range (top and bottom aspects of rectangles), minimum (lowest dot), and maximum (highest dot) values of various clinicopathologic parameters compared between cats, each represented by one dot, with DKA and those with HHS. The HHS group had lower hematocrit and USG, and higher BUN, creatinine, potassium, calcium, magnesium, osmolality, and effective osmolality than the DKA group. DKA, diabetic ketoacidosis; HHS, hyperosmolar hyperglycemic state; Osm, osmolality; USG, urine specific gravity.

The clinicopathologic data of the DKA and HHS groups are summarized in Table 2 and displayed graphically for comparison in Figures 1 and 2.

**TABLE 2:** Summary of clinicopathologic data expressed as median (IQR) for 71 cats with DKA and 29 cats with HHS.

Analyte	Reference interval	DKA	HHS	*p*‐value
Hematocrit	**0.295–0.47 L/L** **29.5%–47%**	**0.336 L/L (0.302–0.366)** **33.6% (30.2–36.6)**	**0.271 L/L (0.23–0.301)** **27.1% (23–30.1)**	**0.0004**
WBC	1.83–16.27 × 10^9^/L 1.83–16.27 × 10^3^/µL	15.9 × 10^9^/L (12.2–22.6) 15.9 × 10^3^/µL (12.2–22.6)	13.8 × 10^9^/L (10.2–18.2) 13.8 × 10^3^/µL (10.2–18.2)	0.32
BUN	4.28–13.9 mmol/L 12–39 mg/dL	**18.21 mmol/L (8.9–27.5)** **51.0 mg/dL (25–77)**	**50 mmol/L (37.5–65.7)** **140 mg/dL (105–184)**	**<0.0001**
Creatinine	53.04–185.64 µmol/L 0.6–2.1 mg/dL	**132.6 µmol/L (106.1–247.5)** **1.5 mg/dL (1.2–2.8)**	**442 µmol/L (247.5–671.8)** **5 mg/dL (2.8‐ 7.6)**	**<0.0001**
Sodium	147–158 mEq/L 147–158 mmol/L	146 mEq/L (139–151) 146 mmol/L (139–151	146 mEq/L (139–150) 146 mmol/L (139–150)	0.91
Potassium	3.9–5.3 mEq/L 3.9–5.3 mmol/L	**3.5 mEq/L (2.9–4.1)** **3.6 mmol/L (2.9–4.1)**	**4.3 mEq/L (3.7–5.1)** **4.3 mmol/L (3.7–5.1)**	**0.0038**
Chloride	113–123 mEq/L 113–123 mmol/L	102 mEq/L (94–110) 102 mmol/L (94–110)	106 mEq/L (100–111) 106 mmol/L (100–111)	0.31
Phosphorus	1.07–2.52 mmol/L 3.3–7.8 mg/dL	**1.31 mmol/L (0.96–1.82)** **4.05 mg/dL (2.98–5.62)**	**3.0 mmol/L (2.26–4.52)** **9.3 mg/dL (7–14)**	**<0.0001**
Total calcium	2.08–2.73 mmol/L 8.3–10.9 mg/dL	**2.23 mmol/L (2.01–2.4)** **8.9 mg/dL (8.05–9.6)**	**2.32 mmol/L (2.17–2.58)** **9.65 mg/dL (8.67–10.3)**	**0.0034**
Magnesium	0.66–0.99 mmol/L 1.6–2.4 mg/dL	**0.99 mmol/L (0.82–1.27)** **2.4 mg/dL (2.0–3.1)**	**1.34 mmol/L (0.97–1.6)** **3.25 mg/dL (2.35–4.07)**	**0.015**
Total bilirubin	0–5.13 µmo*l*/L 0–0.3 mg/dL	**10.3 µmol/L (5.13–27.36)** **0.6 mg/dL (0.3–1.6)**	**3.42 µmol/L (3.42–10.3)** **0.2 mg/dL (0.2–0.6)**	**0.028**
ALP	2–88 U/L	43 U/L (26–71)	52.5 U/L (38.5–79)	0.32
GGT	0–3 U/L	3 U/L (3–3)	3.0 U/L (3–3)	0.93
ALT	16–127 U/L	164 U/L (103–258)	106 U/L (65.2–196)	0.13
AST	14–42 U/L	119 U/L (60–186)	81 U/L (36–164)	0.13
Osmolality	298–319 mOsm/kg	**326 mOsm/kg (308–353)**	**386 mOsm/kg (362–403)**	**<0.0001**
Effective osmolality	275–295 mOsm/kg	**316 mOsm/kg (305–331)**	**337 mOsm/kg (326–347)**	**0.003**
Glucose	4.12–7.93 mmol/L 74–143 mg/dL	**22.8 mmol/L (18.0–34.6)** **411 mg/dL (324–624)**	**43.5 mmol/L (38.9–49.8)** **784 mg/dL (700–897)**	**<0.0001**
Total protein	59–82 g/L 5.9–8.2 g/dL	71 g/L (65–76) 7.1 g/dL (6.5–7.6)	71 g/L (66–82) 7.1 g/dL (6.6–8.2)	0.13
Albumin	24–42 g/L 2.4–4.2 g/dL	31 g/L (27–34) 3.1 g/dL (2.7–3.4)	31.0 g/L (28–34) 3.1 g/dL (2.8–3.4)	1.0
USG	1.010–1.057	**1.029 (1.019–1.040)**	**1.020 (1.016–1.022)**	**0.024**

Abbreviations: ALP, alkaline phosphatase; ALT, alanine transaminase; AST, aspartate aminotransferase; BCS, body condition score; DKA, diabetic ketoacidosis; GGT, gamma‐glutamyl transferase; HHS, hyperglycemic hyperosmolar state; IQR, interquartile range; USG, urine specific gravity.

Significant differences are **bolded**.

*p*‐value represents a *p*‐value with Benjamini–Hochberg correction.

### 3.5 Survival

The overall survival of the combined group was 68% (68/100). No difference was found in survival to discharge between the DKA group (69%, 49/71) and the HHS group (66%, 19/29, *p* = 0.85). Of the 22 DKA cats that did not survive, 13 (59%) were euthanized due to poor prognosis and nine (41%) died naturally. Of the 11 HHS cats that did not survive, eight (73%) were euthanized due to poor prognosis, for two (18%) the manner of death was unknown, and one (9%) died naturally.

No association was found between BCS and survival to discharge for the HHS group (OR = 0.31, 95% CI: 0.07–0.84; *p* = 0.26), the DKA group (OR = 0.81, 95% CI: 0.4–1.61, *p* = 0.66), or the combined group of cats (OR = 0.58, 95% CI = 0.33–1.0; *p* = 0.09). Normal temperature was associated with an increased chance of survival in the DKA group (OR = 2.26, 95% CI: 1.3–4.4; *p* = 0.014) and the combined group (OR = 2.18, 95% CI: 1.33–3.9, *p* = 0.0098), but not in the HHS group (OR = 1.95, 95% CI: 0.69–8.8; *p* = 0.39) (Figure 1; Table 3).

**TABLE 3:** Relationships between various historical, physical examination, and biochemical parameters and survival to hospital discharge for 71 cats with DKA, 29 cats with HHS, and all 100 cats combined.

	DKA: OR (95% CI) for survival	*p*‐value	HHS: OR (95% CI) for survival	*p*‐value	Combined group: OR (95% CI) for survival	*p*‐value
Normal temperature	**2.24 (1.29–4.3)**	**0.014**	1.95 (0.69–8.8)	0.39	**2.18 (1.33–3.9)**	**0.0098**
Abnormal BCS	0.81 (0.40–1.61)	0.66	0.31 (0.07–0.84)	0.26	0.58 (0.33–1.0)	0.09
Prior use of glargine U‐100	2.61 (0.82–10.1)	0.19	1.85 (0.33–14.8)	0.54	2.36 (0.9–7.1)	0.13
Concurrent disease	0.93 (0.33–2.73)	0.89	2.20 (0.38–13.4)	0.44	1.14 (0.47–2.78)	0.78
Increased BUN	**0.25 (0.10–0.56)**	**0.0039**	0.49 (0.16–1.28)	0.35	**0.53 (0.33–0.81)**	**0.014**
Increased creatinine	**0.31 (0.14–0.62)**	**0.0036**	0.50 (0.16–1.23)	0.35	**0.48 (0.29–0.75)**	**0.0098**
Decreased sodium	1.83 (0.99–3.7)	0.11	1.89 (0.91–5.4)	0.35	**1.86 (1.14–3.3)**	**0.03**
Abnormal potassium	0.64 (0.36–1.12)	0.18	0.84 (0.38–1.75)	0.64	0.72 (0.46–1.09)	0.16
Increased phosphorus	**0.23 (0.06‐ 0.71)**	**0.023**	0.60 (0.24–1.22)	0.35	0.62 (0.37–0.98)	0.09
Increased osmolality	0.64 (0.33–1.19)	0.19	0.58 (0.16–1.93)	0.44	0.72 (0.46–1.11)	0.16
Increased effective osmolality	1.08 (0.58–2.02)	0.88	1.44 (0.69–3.5)	0.44	1.13 (0.74–1.78)	0.62
Increased glucose	**0.41 (0.20–0.77)**	**0.016**	0.33 (0.08–1.04)	0.35	**0.53 (0.33–0.83)**	**0.015**

Abbreviations: BCS, body condition score; CI, confidence interval; DKA, diabetic ketoacidosis; HHS, hyperglycemic hyperosmolar state; OR, odds ratio.

Significant results are **bolded**.

*p*‐value represents a *p*‐value with Benjamini–Hochberg correction.

The presence of concurrent disease was not associated with odds of survival in any group (DKA group: OR = 0.93, 95% CI: 0.33–2.73; *p* = 0.89; HHS group: OR = 2.2, 95% CI: 0.38–13.4; *p* = 0.44; combined group: OR = 1.4, 95% CI: 0.47–2.78, *p* = 0.78). Similarly, the use of glargine U‐100^a^ was not associated with survival in any group (DKA: 81.8% vs. 63.3%, OR = 2.61, 95% CI: 0.82–10.1; *p* = 0.19; HHS: 75% vs. 61.9%, OR = 1.85, 95% CI: 0.33–14.8; *p* = 0.54; combined: OR: 2.36, 95% CI: 0.9–7.1, *p* = 0.13).

In the DKA group, higher serum BUN (*p* = 0.0039), creatinine (*p* = 0.0036), phosphorus (*p* = 0.023), and BG (*p* = 0.016) concentrations were associated with an increased risk of death (Table 3; Figure 1). In the HHS group, however, no biochemical variables were associated with survival (Table 3; Figure 1). In the combined group, higher serum BUN, creatinine, and glucose concentrations and lower serum sodium concentration were associated with decreased odds of survival in the univariate analysis (Table 3; Figure 1).

## 4 Discussion

The current study compared various clinical parameters between cats with HHS and those with DKA, evaluated for association between these parameters and survival to discharge in each group and in the combined group of 100 cats experiencing hyperglycemic diabetic crisis, and compared survival outcome for each group. Survival to discharge for cats with HHS was more common in this population of cats than previously reported [1] and was not different than survival in cats with DKA. No presenting physical examination characteristic or blood work values were associated with survival in the HHS group, and prior use of glargine U‐100^a^ was not associated with survival in any group of cats.

The most comprehensive study to date of feline HHS reported survival of 35.3% [1]. This low survival may influence clinical decisions for both clinicians and clients, as the willingness to treat a condition with a poor reported outcome may be less likely. This study found a higher absolute survival of 65.5% in the HHS population, though this comparison was not analyzed statistically. Possibly the larger sample size in this study of 29 cats compared to the previous study by Koenig et al. [1] involving 17 cats may be more representative of HHS in cats. The survival found in the current study may be attributed to several factors, such as increased owner willingness to treat, different population of cats, or differences in clinician treatments and management of diabetic cats. The current study still has a small sample size, and further studies are warranted to explore the potential for good outcomes for cats with HHS. Interestingly, the survival found in the current study is similar to the 62% survival reported in dogs diagnosed with HHS [3]. The survival from HHS in people ranges from 50% to 90%, with younger patients having a worse prognosis [16]. In the current study, survival in the DKA group was 69%, which is subjectively consistent with the previously reported survival in feline DKA of 69%–100% [2, 4, 17].

As previously stated, 25%–30% of human patients presenting in a hyperglycemic diabetic crisis have combined features of DKA and HHS, and separating these syndromes is considered arbitrary due to their similar signs, underlying pathophysiology, and treatment regimens [7, 16, 18]. The similar survival found in the current study between DKA and HHS further supports that these syndromes overlap and may be a continuum of manifestations of critical illness in diabetic cats. This outlook could increase the support for pursuing treatment rather than euthanasia in cats with HHS. Attempting to treat more cats with HHS would allow the veterinary community to explore further the survival potential of this condition.

Another key finding of the current study was the lack of association between osmolality and survival in any group. Median total and effective calculated osmolalities were higher in the HHS group than in the DKA group, as expected based on the criteria used to group the cats. It is believed that kidney injury that impairs glucose excretion due to decreased GFR contributes to the development of HHS [4, 10, 11, 17]. Interestingly, neither total nor effective osmolality was associated with survival in any group and showed considerable overlap between the HHS and DKA groups; this finding supports that the separation of HHS and DKA in veterinary medicine may be arbitrary.

In this study, decreased body temperature was associated with survival in the DKA and combined groups, but not the HHS group. People with DM have impaired vasomotion, which is presumed to facilitate peripheral perfusion [19]. Cats with DM may also have decreased vasomotion and reduced perfusion, which could lead to hypothermia. Additionally, a state of shock could contribute to hypothermia. In this study, the lack of association between temperature and survival in the HHS group may reflect a type II error considering the small number of patients in the HHS group, because this inter‐group difference is unexpected. It is reasonable to use presenting temperature as a prognostic indicator since hypothermia has been associated with decreased survival in many conditions in cats, including trauma, uremia, sepsis, and pancreatitis [20, 21, 22, 23]. A lower temperature's association with poor outcome in these conditions likely reflects greater severity of illness, worsened hemodynamic compromise, and shock, which can cause hypothermia in cats [23]. Our findings support the presence of decreased body temperature as a negative prognostic indicator for survival in DKA and the overall population of sick, hyperglycemic diabetic cats. The use of temperature as a prognostic indicator for cats with HHS requires further investigation.

The current study found that cats in the HHS group were older than those in the DKA group and were more likely to have an underlying chronic disease, with kidney disease being the most prevalent. These findings are likely related since chronic kidney disease is common in older cats and likely contributes to the development of HHS due to poor GFR. This is similar to findings of previous studies that found that kidney disease and congestive heart failure are predisposing factors for development of HHS, as both conditions are associated with reduced GFR [1, 10]. An impaired ability to renally excrete glucose can result in severe hyperglycemia and hyperosmolality, which may increase the risk of developing HHS rather than DKA in cats with underlying kidney disease.

In the DKA and combined groups, higher serum BUN and creatinine concentrations were associated with decreased survival. The HHS group had a higher degree of azotemia than the DKA group; however, no association with survival was found in the HHS group. Clinically, degree of azotemia is important because it can provide insight into the severity of dehydration, acute kidney injury (AKI), or underlying chronic kidney disease [1, 10, 24, 25]. The higher degree of azotemia in HHS cats may explain why this group had lower median hematocrit and lower median urine specific gravity compared to the DKA group. It is also possible that the hyperosmolality led to water shifting into the extracellular fluid compartment, diluting the hematocrit. It is plausible some clinicians may alter their clinical recommendations and therapy based on the degree of azotemia, which ultimately may impact an owner's decision to pursue euthanasia rather than treatment. In this study, however, azotemia was not associated with a lower OR of survival in the HHS group. This may again be due to the small sample size, but it could also be explained by the fact that cats with HHS are more likely to have underlying kidney disease, higher baseline kidney values, and a more significant fluid‐responsive azotemia [1, 26]. An equivalent degree of azotemia in a cat with DKA or sick diabetic cat without underlying kidney disease could reflect a more dramatic change from the animal's baseline kidney values, thus indicating a more meaningful AKI and maybe a worse prognosis [4, 25, 26, 27]. Overall, the findings of this study support the presence of azotemia being associated with decreased survival in DKA and diabetic cats undergoing a diabetic crisis, while the association of azotemia with survival for cats specifically diagnosed with HHS requires further investigation.

Serum phosphorus concentration was also higher in the HHS group than in the DKA group. In this study, no association was found between serum phosphorus concentration and survival in the HHS or combined groups, but higher phosphorus was associated with decreased survival in the DKA group. Similarly, Koenig and colleagues [1] reported a significantly higher serum phosphorus concentration in their HHS group compared to cats with DKA. The higher phosphorus value in the HHS group may be explained by a longer onset of disease and a prolonged timeframe without insulin or underlying insulin resistance [4, 28] or by underlying kidney disease and poor GFR [29, 30]. The association between hyperphosphatemia and survival in the DKA group could also be related to an acute onset of disease, a more severe AKI with a reduction in GFR, or a more significant change from baseline phosphorus concentrations. Recently, hyperphosphatemia has been associated with decreased survival from critical illness in human medicine, with worsened prognosis associated with a more severe increase in serum phosphorus [31]. Lastly, it is possible that a larger sample size would reveal an association between survival and increased phosphorus concentration in the HHS and combined groups.

A decreased serum sodium concentration was associated with decreased odds of survival in the combined group, but not in the DKA or HHS groups. Physiologically, it is plausible that hyponatremia could be secondary to increased renal sodium wasting from severe osmotic diuresis, transcellular water shifting from hyperosmolality and hyperglycemia, and increased renin–aldosterone–angiotensin system activation [12, 24]. Overall, the current study supports the use of hyponatremia as a negative prognostic indicator for cats undergoing a hyperglycemic diabetic crisis. However, further investigation into serum sodium concentration and survival in the specific conditions of DKA and HHS cats is warranted, as the serum sodium evaluated was not adjusted for hyperglycemia. It is possible this association could be driven by the degree of hyperglycemia.

Worsened hyperglycemia was only associated with decreased survival in the DKA and combined groups, but not the HHS group. This discrepancy may be due to the smaller population of cats with HHS compared to those with DKA, and a larger population may have reached statistical significance in the HHS group. Hyperglycemia's association with survival in the DKA and combined groups may reflect more severe underlying disease and increased cortisol secretion, with cortisol being a potent diabetogenic hormone that exacerbates insulin resistance [4, 10]. Further studies are needed to evaluate this association with a larger population.

Administration of glargine U‐100^a^ was not associated with survival in any group in the current study. Although insulin types were noted in the Koenig et al. [1] study, glargine U‐100^a^ was not mentioned, likely because it was not commonly used in veterinary medicine at that time. It is possible that the type of previously prescribed insulin does not affect survival of an animal in a diabetic crisis based on the underlying pathophysiology leading to crisis development. However, with a larger sample size, it is possible that a relationship between glargine U‐100^a^ and survival could be elucidated. Therefore, additional clinical investigation is warranted to further clarify glargine U‐100's^a^ potential role in feline diabetic remission and recovery from diabetic crises.

There are several limitations to this study. Its retrospective nature prevented standardization of diagnostic tests, treatments, and clinical decisions, which could have affected survival to discharge. Additionally, the small sample size for the HHS group likely led to underpowering of this group and decreased the chance for associations to be found for this group. Another limitation is the inclusion criteria chosen for the HHS group; definitions of HHS in the veterinary literature vary, as some sources define the state with a BG cutoff >600 mg/dL and total osmolality >350 mOsm/kg, while other sources use a BG cutoff >540 mg/dL and total osmolality >330 mOsm/kg [1, 4, 10, 15, 17]. The investigators selected a value between the previous studies’ parameters for the current study's HHS definition to include a larger group of cats. Thus, BG >560 mg/dL with total osmolality >350 mOsm/kg along with nonketosis were used to assign an HHS group designation, which may have contributed to the possible survival difference compared to previous reports. Additionally, calculated (rather than measured) total osmolality was used as part of the inclusion criteria for the HHS group. Also, there was a lack of standardization for the measurement of ketosis. A combination of measured serum beta‐hydroxybutyrate concentrations and urine dipsticks for presence of acetoacetic acid was used to evaluate for the presence of ketosis. It has been established that urine dipstick tests are sensitive to the presence of acetoacetic acid; however, false positives and negatives can occur. The measurement of serum ketones is considered superior for the detection of ketones [32, 33]. Therefore, it is possible some patients could have been categorized incorrectly in either group if a urine dipstick was used. Including euthanized cats in the outcome analysis is a limitation because clinicians may have altered treatment recommendations based on perceived differences in prognosis for DKA versus HHS. Also, it is impossible to understand fully the influences that finances, prognosis, and other personal factors carry when clients have an option to euthanize a critically ill pet, regardless of disease or study design. A final limitation was the inability to perform a meaningful multivariate analysis due to the size and characteristics of the dataset.

In conclusion, cats with HHS had a subjectively better survival of 65.5% in the current study compared to that previously reported [1]. No specific negative clinical prognostic indicators were identified for cats with HHS compared to those with DKA, and insulin type was not associated with survival in any group. This study revealed that generalized indicators for worse prognosis in sick diabetic cats included decreased body temperature, worse azotemia, degree of hyperglycemia, and hyponatremia.

## Author Contributions

**Annalisa Judy**: data curation, formal analysis, investigation, project administration, writing – original draft, writing – review and editing. **Aaron Rendahl**: data curation, formal analysis, methodology, visualization, writing – review and editing. **Kelly Tart**: conceptualization, formal analysis, project administration, supervision, writing – original draft, writing – review and editing.

## Conflicts of Interest

The authors declare no conflicts of interest.

## Endnotes

a Glargine U100, Boehringer Ingelheim Pharmaceuticals Inc, Ingelheim am Rhein, Germany.

b ADVIA 2120i, Siemens, Munich, Germany.

c AU480, Beckman Coulter, Brea, CA.

d iStat, Abbott Laboratories, Abbott Park, IL.

e ProZinc, Boehringer Ingelheim Pharmaceuticals Inc, Ingelheim am Rhein, Germany.

f Lente, Eli Lilly & Co, Indianapolis, IN.

g Vetsulin, Merck Animal Health, Rahway, NJ.

h Neutral Protamine Hagedorn, Eli Lilly & Co, Indianapolis, IN.
